# Engineering Poly(L-Lactic Acid)/Hydroxyapatite Scaffolds via Melt-Electrowriting: Enhancement of Osteochondral Cell Response in Human Nasal Chondrocytes

**DOI:** 10.3390/polym17182455

**Published:** 2025-09-10

**Authors:** Valentina Basoli, Vittorio Barbano, Cecilia Bärtschi, Cosimo Loffreda, Matteo Zanocco, Alfredo Rondinella, Alex Lanzutti, Wenliang Zhu, Stefania Specchia, Andrea Barbero, Florian Markus Thieringer, Huaizhong Xu, Elia Marin

**Affiliations:** 1 Medical Additive Manufacturing Research Group (Swiss MAM), Department of Biomedical Engineering, University of Basel, Hegenheimermattweg 167C, 4123 Allschwil, Switzerland; cecilia.baertschi@unibas.ch (C.B.); cosimo.loffreda@unibas.ch (C.L.); florian.thieringer@usb.ch (F.M.T.); 2 Ceramic Physics Laboratory, Faculty of Materials Science and Engineering, Kyoto Institute of Technology, Sakyo-ku, Matsugasaki, Kyoto 606-8585, Japan; vbarbano97@gmail.com (V.B.); wlzhu@kit.ac.jp (W.Z.); 3 Politecnico di Torino, Department of Applied Science and Technology, Corso Duca degli Abruzzi 24, 10129 Torino, Italy; stefania.specchia@polito.it; 4 Department Polytechnic of Engineering and Architecture, University of Udine, Via delle Scienze, 206, 33100 Udine, Italy; matteo.zanocco@uniud.it (M.Z.); alfredo.rondinella@uniud.it (A.R.); alex.lanzutti@uniud.it (A.L.); 5 Department of Biomedicine, University of Basel, University Hospital Basel, Hebelstrasse 20, 4031 Basel, Switzerland; andrea.barbero@usb.ch; 6 Clinic of Oral and Cranio-Maxillofacial Surgery, University Hospital Basel, 4031 Basel, Switzerland; 7 Department of Biobased Materials Science, Kyoto Institute of Technology, Sakyo-ku, Matsugasaki, Kyoto 606-8585, Japan; xhz2008@126.com; 8 Biomedical Research Center, Kyoto Institute of Technology, Sakyo-ku, Matsugasaki, Kyoto 606-8585, Japan; 9 Materials Innovation Laboratory, Kyoto Institute of Technology, Sakyo-ku, Matsugasaki, Kyoto 606-8585, Japan

**Keywords:** electrospinning, poly(L-lactic acid), bone guide regeneration membrane scaffolds, tissue engineering, cellular proliferation, hydroxyapatite

## Abstract

Osteochondral repair remains challenging due to cartilage’s limited self-healing capacity and the structural complexity of the osteochondral interface, particularly the hypertrophic layer anchoring cartilage to bone. We fabricated melt electrowritten (MEW) poly(L-lactic acid) (PLLA) scaffolds incorporating 1%, 5%, and 10% hydroxyapatite (HAp) to provide a precise fiber architecture (~200 μm pores) and bone-mimetic biochemical cues. Human nasal chondrocytes (hNCs), currently in clinical trials for knee cartilage repair, were selected for their phenotypic plasticity and established safety profile, facilitating translational potential. HAp–PLLA scaffolds, especially at higher HAp contents, enhanced hNC adhesion, proliferation, mineralization, and maintenance of cartilage-specific ECM compared to PLLA alone. This work demonstrates the first high-HAp MEW-printed PLLA scaffold for osteochondral repair, integrating architectural precision with bioactivity in a clinically relevant cell–material system.

## 1. Introduction

Osteochondral repair, poses significant challenges in the field of regenerative medicine [1,2,3,4]. Cartilage has limited intrinsic healing capacity due to its avascular nature and low cell turnover rate [5,6]. Traditional treatments often fail to restore full functionality, highlighting the urgent need for innovative approaches to promote effective tissue repair [7,8]. One of the most intricate aspects of cartilage regeneration is due to the complexity of the tissue, which is characterized by different layers of chondrocytes embedded in extracellular matrix that organize to provide a mechanical structure ensuring the functionality of the cartilage. This is essential for cushioning and reducing friction. Cartilage is a tissue organized in layers: the superficial, intermediate, and deep or hypertrophic layer, which gradually becomes osseous, connecting the underlying bone. This deep layer, caller interphase layer or hypertrophic is fundamental as it forms the foundation for all the upper layers.

In recent years, significant interest has been directed towards the use of cells for cartilage regeneration [9,10,11]. Common clinical use is from cells from adipose tissue, bone marrow, or, as gold standard technique, microfractures that allow bone marrow cells to partially regenerate the cartilage area [12]. Finally, isolated chondrocytes from the nasal septum [13] have shown promise in Phase I and Phase II clinical studies for the regeneration of focal cartilage defects [14,15] identifying them as excellent candidates [13]. From a translational perspective, this is one of the first examples of cells in combination with material. Nasal chondrocytes (hNCs) were isolated from the patients, grown in the Good manufacturing practice (GMP) laboratory, and finally seeded onto a scaffold or, more precisely, a medical device made of collagen sponge [16]. Although this collagen scaffold has good biological properties, it is a membrane with fibers arranged in anisotropic way [17]. Therefore, considering the complexity and importance of osteochondral organization, having a support that mimics natural tissue is of fundamental importance. Indeed, central to successful regeneration is the development of scaffolds capable of supporting proper cellular adhesion, proliferation, and tissue integration [18,19,20].

In the past years, various techniques have been employed to fabricate scaffolds for tissue regeneration, including solvent casting [21,22], electrospinning [23,24], and 3D printing fused deposition modeling (FDM) [25,26]. These methods have shown promise but often face limitations such as poor control over pore structure and limited scalability [27]. Recently, melt-electrowriting (MEW) has emerged as a cutting-edge technique for scaffold fabrication with precise control over architecture and composition [28,29,30]. However, new methods that combine bio-3D printing and melt electrowriting are emerging and show great promise.

MEW utilizes a combination of melt extrusion and electrostatic deposition to create highly porous scaffolds with tailored microstructures. This innovative approach has demonstrated remarkable success in tissue engineering applications, offering advantages in terms of structural fidelity, mechanical properties, and bioactivity. Moreover, the technology behind MEW allows to produce regular scaffolds with porosities in the range of hundreds or tens of microns, which would be incompatible with most other 3D printing technologies on the market [31,32,33,34].

Processing composite biomaterials with MEW is particularly challenging due to the extremely small nozzle diameter and the application of an external electric field between the nozzle and the collector, which makes the process highly sensitive to material properties. The inclusion of ceramic fillers such as hydroxyapatite (HAp) can increase the risks of nozzle clogging, porosity formation, and fiber irregularities due to changes in melt viscosity and electrical resistivity.

Poly(L-lactic acid) (PLLA) has been extensively explored in tissue engineering due to its biocompatibility, biodegradability, and mechanical properties resembling those of native tissues [35,36,37,38]. However, the use of PLLA for biomedical scaffolds present two main limitations: they are prone to UV degradation, limiting the selection of possible sterilization technologies [39] and they present poor cellular adhesion, which compromises tissue integration and regeneration [40].

Despite these drawbacks, PLLA represents one of the most suitable polymers for MEW after polycaprolactone (PCL), whose lower mechanical strength limits its applicability in load-bearing scaffolds. Modern MEW machines can process newer high-performance polymers with potentially superior properties, but these are more difficult to print and are not yet compatible with ceramic fillers such as HAp.

In this study, we address the challenge of enhancing cellular interactions by integrating hydroxyapatite particulates (HAp), an osteoconductive calcium phosphate phase known to provide ionic dissolution products (Ca^2+^, PO_4_^3−^) that regulate integrin-mediated adhesion, focal adhesion kinase signaling, and subsequent osteogenic lineage commitment of stem cells, into PLLA scaffolds. The PLLA/HAp composite scaffolds are expected to improve bioactivity by promoting the differentiation of human nasal chondrocytes (hNC) toward the hypertrophic osteochondral interface [41], while also providing suitable mechanical support for the cells. This makes them promising candidates for osteochondral regeneration.

Additionally, we investigated the use of PLLA/HAp scaffolds fabricated via melt electrowriting (MEW) for osteochondral regeneration applications aiming to achieve a high resolution and miniaturization of micro scaffolds. Therefore, by optimizing the scaffold composition and microstructure, we seek to enhance cellular adhesion, proliferation, and extracellular matrix deposition focusing on the biocompatibility and regenerative potential of these scaffolds in vitro.

PLLA/HAp composite scaffolds have been previously investigated for their enhanced biological properties [42,43,44], and scaffolds have been previously produced using techniques such as or electrospinning [45].

The novelty of this study lies in the integration of these composite with the MEW technology, which allows to create scaffolds with regular porosities and high spatial resolution in order to provide improved biological and mechanical properties for osteochondral regeneration. Moreover, in this study, we investigated the behavior of PLLA scaffolds incorporating a high particulate content of up to 10 wt%. This concentration is significantly higher than what was previously achievable through electrospinning.

We hypothesize that having a material capable of providing a osteogenic/hypertrophic stimulus, due to the presence of HAp, might improve the osteochondral tissue formation at the cartilage-bone interface. Finally, in this work, we have studied how MEW can effectively be used to produce micro-scaffolds, such as meshes that, from a clinical perspective, could potentially be used in the future instead of membranes on which cells can be seeded, or simply as scaffolds to be used in the area of focal defects to drive underlying cells from the bone.

## 2. Materials and Methods

### 2.1. Composite Materials

Polylactic acid (PLLA) pellets (2.5 g) were weighed along with hydroxyapatite (HAp) in predetermined fractions 0.025 g for 1 wt.%, 0.125 g for 5 wt.%, and 0.25 g for 10 wt.% and placed in a 50 mL Falcon tube. A chloroform solution was used to complete dissolution of PLLA. The mixture was subjected to agitation at 280 rpm and 40 °C for 4 h using a magnetic stirrer, with the vial initially sealed to minimize chloroform evaporation.

Simultaneously, HAp powder was dispersed in chloroform in a separate 10 mL vial, which was filled to approximately 50% capacity. To achieve uniform dispersion, the vial was placed in a water bath within a bath sonicator and sonicated for 4 h.

Following dispersion, the HAp suspension was gradually introduced into the PLLA solution under continuous stirring. The vial was left open to facilitate chloroform evaporation. To accelerate solvent removal and enhance the homogenization of HAp nanoparticles, the temperature was increased to 70 °C, and the stirring speed was raised to 360 rpm. The mixture was stirred for approximately 1 h or until roughly 50% of the chloroform had evaporated.

The resulting solution was cast into a Petri dish and allowed to dry overnight in a ventilated chamber at ambient temperature and pressure. The dried composite film was subsequently cut into small rectangular specimens and subjected to further drying under vacuum at 110 °C for 2 h, followed by additional drying at 80 °C for 6 h to ensure complete solvent removal.

### 2.2. Melt-Electrowriting

The detailed description of the Melt-electrowriting equipment and the process parameters has been previously published elsewhere [28,29].

The collector surface temperature was maintained at 80 °C to minimize thermal shock upon filament deposition, thereby ensuring optimal adhesion during initial contact. The print head temperature was set to 190 °C, slightly above the melting point of poly(L-lactic acid) (PLLA), yet within a controlled range to mitigate thermal degradation a critical factor affecting scaffold structural integrity. This thermally controlled environment facilitated the continuous fabrication of multiple scaffolds within a single session.

A 30-gauge needle was integrated with a customized metallic syringe connected to a pressure control unit, enabling precise and continuous polymer extrusion within a pressure range of 0.02 to 0.2 MPa. A high-voltage electric field was applied, with an intensity set between 2.0 and 3.5 kV, maintaining a 2.5 mm gap between the syringe tip and the collector surface to ensure controlled fiber deposition.

Each printing cycle was completed after fabricating between 5 and 20 scaffolds. The scaffold architecture featured a square grid pattern with 200 µm interfilament spacing, consisting of 25 parallel filaments in both horizontal and vertical orientations. The constructs were printed in 25 layers, forming a final scaffold structure with a surface area of 1 cm^2^.

### 2.3. Characterization Techniques

#### 2.3.1. Confocal Imaging

Micrographs of the scaffolds were taken using a 3D laser-scanning microscope (VKX200K series, Keyence, Osaka, Japan) with magnifications ranging from 10× to 150× and a numerical aperture between 0.30 and 0.95. Surface maps obtained from merging of different images were acquired by using a dedicated automated xy stage combined with the autofocus function for the z axis.

#### 2.3.2. Scanning Electron Microscope

A SM-700 1F Scanning Electron Microscopy (JEOL, Tokyo, Japan) was employed to capture high-magnification images of the scaffolds both pre- and post-biological testing. Prior to observation, the samples underwent sputter-coating with a platinum layer (approximately 2 nm thick) and were subsequently examined at an accelerating voltage of 10 kV.

#### 2.3.3. Raman Spectroscopy

Raman imaging was conducted utilizing a confocal Laser Raman microscope (RAMANtouch, Nanophoton Co., Ltd., Osaka, Japan) with excitation sources at 532 nm and a nominal power of 200 mW. To mitigate the risk of sample burning, the power output was regulated by adjusting a dedicated ND filter. The micro-probe employed lenses ranging from 5× to 100× magnifications, with numerical apertures spanning from 0.5 to 0.23. Imaging involved the acquisition of linear arrays (x axis) consisting of 400 points, which were subsequently combined into a bi-dimensional map (y axis).

The average spectra for each material were then analyzed and deconvoluted using a dedicated software (Labspec 5.0, Horiba, Kyoto, Japan).

#### 2.3.4. X-ray Diffraction

XRD analyses were performed on a Rigaku Ultima IV (Rigaku Corporation, Tokyo, Japan), using the CuKa radiation. Diffraction patterns were acquired in the range 5–50° with a step size of 0.02 at a rate of 3°/min. The penetration depth of the XRD probe was in the order of 1 mm. For PLLA, the peaks at about 17° (110), 19° (203) and 32° (200) [46,47,48,49] were considered representative of the crystalline phase, while the main peaks related to HAp could be found at about 26°, 29°, 32° and 34° [47,48,49]. The crystallinity index was calculated as the ratio between the integrated intensity of the crystalline peaks and the total integrated intensity after subtraction of the HAp contribution, following the equation:

$$X_{c}=\frac{I_{PLLA}}{I_{total}-I_{HAp}}\times 100$$

where $X_{c}$ is the crystallinity index, $I_{PLLA}$ is the sum of the intensity of the two main peaks related to PLLA, $I_{HAp}$ is the cumulative intensity of the peaks related to HAp and $I_{total}$ is the total integrated intensity.

XRD analyses were performed on both the raw materials before MEW (in sheet form) and the 3D printed scaffolds.

#### 2.3.5. Thermogravimetric Analysis

The thermal stability of PLA and the composites was evaluated by mean of Thermogravimetric Analysis. The samples were heated in air atmosphere from room temperature to 600 °C with 10 °C min^−1^ heating rate under nitrogen atmosphere (platinum crucible) in a Thermo plus TG 8120 (Rigaku, Tokyo, Japan). Thermogravimetric analyses were performed on both the raw materials before MEW (in sheet form) and the 3D printed scaffolds. A small section of each scaffold or sheet was carefully cut to fit the TGA sample holder for analysis.

### 2.4. Biological Testing

#### 2.4.1. Cell Culture for Toxicity Evaluation

BJ-1 cells were cultured at a seeding density of 3 × 10^3^ cells·cm^−2^ in DMEM (Dulbecco’s Modified Eagle Medium, High Glucose) supplemented with 10% Fetal Bovine Serum (FBS, Gibco, Thermo Fisher, Zürich, Switzerland), 100 U/mL penicillin, and 100 μg·mL^−1^ streptomycin (Gibco, Thermo Fisher, Zürich, Switzerland). The medium was refreshed every second day until the confluence of 70–80%. BJ-1 cells were maintained at 37 °C in a humidified atmosphere with 5% CO_2_.

#### 2.4.2. Cell Culture for Osteochondral Interface Differentiation

Nasal Chondrocytes (hNCs) isolated from anonymized donor nasal septum cartilage were cultured maintaining a seeding density of 3 × 10^3^ cells·cm^−2^ using expansion medium made of Dulbecco’s Modified Eagle Medium (DMEM, Gibco, Thermo Fisher, Zürich, Switzerland) with 5% FBS, 100 U·mL^−1^ penicillin, 100 μg·mL^−1^ streptomycin (Gibco, Thermo Fisher, Zürich, Switzerland), 1 ng·mL^−1^ Transforming Growth Factor-beta1 (TGF-β1, Biotechne, Minneapolis, MN, USA) 5 ng·mL^−1^ Fibroblast Growth factor-2 (FGF-2, Biotechne). Medium was changed every second day. hNC cells were maintained at 37 °C in a 5% CO_2_ humidified atmosphere until reaches the confluency of 70–80%. hNC were used between passage 3 and 5.

Expanded hNCs were then detached and seeded onto a PLLA-modified scaffold in a TC24 well that had been pre-coated with 1.5% agarose gel to prevent cell adhesion to the tissue culture plate and incubated with expansion medium. After 24 h, the expansion medium was replaced with a medium composed of Minimal Modified Eagle Medium (α-MEM, Gibco, Thermo Fisher, Zürich, Switzerland) with 10% FBS, 1% HEPES (Gibco, Thermo Fisher, Zürich, Switzerland), and 100 U·ml−1 penicillin, 100 μg·ml^−1^ streptomycin, named as Basic Medium (BM) (Gibco, Thermo Fisher, Zürich, Switzerland). To enhance osteogenic differentiation and matrix mineralization, BM was supplemented with 10 nM dexamethasone (Sigma, Buch, Switzerland) and 100 μM ascorbic acid-2 phosphate (Sigma, Buch, Switzerland) and 10 mM Glycerophosphate (Gibco, Thermo Fisher, Zürich, Switzerland) (to generate the Osteogenic medium, OM).

#### 2.4.3. Metabolic Activity and Indirect Toxicity Test

Cell Titer Blue (Promega, Dübendorf, Switzerland) was used to assess metabolic activity at 24 and 72 h in cells exposed or unexposed (live CTR cells) for 24 h to PLLA+ Hydroxy Apatite. Specifically, the PLLA + 1% HA, PLLA + 5% HA, and PLLA + 10% HAp were immersed in 1 mL of cell culture medium for 24 h, according to ISO 10993:5 [50] guidelines. To the cells already adherent in TC24 wells (BJ-1) medium containing the test material was replaced after 24 h. Following 24 or 72 h of exposure to the material solution, the medium was aspirated, and 300 µL of medium along with Cell Titer solution were added per well according to the manufacturer’s instructions. After 2 h, the medium contained the metabolized cell titer blue was transferred into a 96-well and the fluorescent signal was measured at λex_560_/λem_590_ nm. A working solution was used as the minimally viable control (Blank), with wells blanked using the average medium fluorescence. Metabolic activity was determined by calculating the percentage of control cells not treated. Control dead cells were treated with pure DMSO for 15 min.

#### 2.4.4. Nasal Chondrocytes Cell Adhesion and Live and Dead

hNC were tested on PLLA modified material. In order to avoid the adhesion of cell on tissue culture plate, TC24 well were precoated with 200 µL Agarose 1.5%. After solidification of Agarose, PLLA and PLLA + HAp modified samples were located in the wells. Respectively 250’000 cells in 300 µL were seeded on scaffolds (5 mm × 5 mm) in Basal and Osteogenic Medium. All samples were prepared in triplicates.

hNC cells were co-stained with 5 µM Calcein (Sigma-Aldrich, St. Louis, MO, USA, #17783) and 0.625 µg/mL Ethidium Homodimer for 30 min to discriminate between live and dead cells.

#### 2.4.5. Nasal Chondrocytes Phalloidin Staining (Morphology)

Morphological evaluation of cell organization was performed using phalloidin staining.

Cell and scaffold were fixed using Buffered-Formalin 4% for 20 min (Formafix, Hittnau, Switzerland). After an initial washing in PBS, patterned cells were permeabilized with 0.25% Triton X-100 for 15 min RT, then stained with 2 μg·ml^−1^ of ATTO594-conjugated phalloidin (Sigma-Aldrich, St. Louis, MO, USA, # 51927) for 45 min at RT. DAPI (Bioting, #40009) as nuclei counterstaining was used at 2.5 ug/mL. All samples were prepared in triplicates.

#### 2.4.6. Alizarin Red Staining

To assess the mineralization potential of the nasal chondrocytes cultured in the melt electrowritten scaffolds PLLA modified with HA, alizarin red S staining of calcium deposited in the extracellular matrix of nasal chondrocytes cultured on the scaffolds after 21 days was carried out. Briefly, the culture media was removed and the scaffolds washed using PBS. The cells on the scaffolds were fixed in Buffered-Formalin 4% (Formafix, Hittnau, Switzerland), wash again in PBS and then placed in 300 μL of Alizarin Red S solution (Sigma, Buch, Switzerland, #A5533-25G) at 40 mM for 20 minutes at room temperature. After washing in distilled water two times, the stained nasal chondrocytes and scaffolds were imaged using a stereoscope DVM6 digital microscope (LEICA microsystems, Wetzlar, Germany) at magnification 50× and 200×. Subsequently, the quantification of calcium deposition was achieved by dissolving the samples in 1 mL 10% Cetylpyridinium Chloride (Sigma, Buch, Switzerland, #C0732-100G) for 1 h before transferring 100 uL to 96 well plates and measuring the absorbance at 540 nm in a microplate reader (Varioskan LUX, Thermo Fisher, Zürich, Switzerland). The average value of the negative controls (scaffold without cells) was subtracted from the values of the corresponding experimental groups.

#### 2.4.7. Alcian Blue Staining

Samples at day 21 were fixed in Buffered-Formalin 4% (Formafix, Hittnau, Switzerland), washed with 1xPBS, 200 µL of 3% acetic acid was added for 1 min. After removing the acetic acid, 200 µL of Alcian Blue (Sigma, Buch, Switzerland, # TMS-010) staining reagent was added and incubated for 45 min at room temperature. Subsequently, the samples were washed with milliQ water for three times and left in 1xPBS. Images were then taken using DVM6 digital microscope (LEICA microsystems) to visualize the entire well and AE31 inverted microscope (Motik, Xiamen, China) to capture more detailed images. To quantify the amount of Alcian Blue retained in the proteoglycan-rich matrix, samples were incubated overnight in 6M guanidine hydrochloride (GdnHCl) solution (Sigma, Buch, Switzerland, #50950). Guanidine hydrochloride, a strong protein denaturant, facilitates the solubilization of the Alcian Blue-stained matrix, enabling subsequent spectrophotometric measurement. The extracted dye concentration was determined by measuring 100 µL of solubilized solution using absorbance reading at 595 nm, providing a quantitative readout of proteoglycan content.

#### 2.4.8. Immunofluorescence

Samples were processed for immunofluorescent labeling according to a standardized protocol. Initially, permeabilization was performed using Triton X-100 solution (Life Technologies, Carlsbad, CA, USA) for 15 min at room temperature. This was followed by a blocking step with 1% bovine serum albumin (BSA) in phosphate-buffered saline (PBS) for 15 min to minimize nonspecific binding. Following blocking, samples were incubated overnight at 4 °C in BSA-containing solution supplemented with the following primary antibodies: anti-Collagen X (1:50) (abcam, Cambridge, UK, #AB49945, After primary antibody incubation, samples were washed three times with 500 μL of PBS to remove unbound antibodies. Secondary antibodies anti-mouse Alexa Fluor 546 (Invitrogen, A21123, Waltham, MA, USA) were applied at a concentration of 5 μg/mL (Imaging was performed using a Nikon AXR-GH confocal microscope).

### 2.5. Imaging Analysis

Phase-contrast and fluorescence images for morphology (phalloidin) were acquired using a Nikon AXRGH confocal microscope (Nikon Corporation, Tokyo, Japan) equipped with a CCD camera at magnification 10× (0.16), 20× (water immersion objective 0.8).

Phase-contrast and fluorescence images for live and dead and Alcian blue were acquired using a EVOS M5000 (Thermo Scientific, Switzerland) equipped with a CCD camera at magnification 10× (0.16), 20× (water immersion objective 0.8).

The brightness of predefined regions of interest (ROIs) in multi-channel .nd2 files was analyzed using a custom macro in ImageJ (ImageJ 1.54f, National Institutes of Health, Bethesda, MD, USA) with Bio-Formats (version 6.13.0, Open Microscopy Environment, University of Dundee & Glencoe Software, Inc., Dundee, UK) for file handling. The macro iterated through all *.nd2 files within a selected folder, loading each image using Bio-Formats Importer to access the multi-dimensional data, where each file contained multiple channels, with each channel corresponding to antibody staining conditions. For each image, the macro first increased the brightness of all channels by 50% using the “Multiply” function to adjust for possible image underexposure. It then defined five square regions (500 × 500 pixels each) at specific positions specifically set close to the center of the image to accommodate the scaffold location: center, center top-left, center top-right, center bottom-left, and center bottom-right. The mean brightness within each of these ROIs was measured for each channel in the image. In ImageJ, brightness is measured by calculating the mean pixel intensity within the selected ROI. The pixel intensity range for 16-bit images is from 0 to 65,535, so the measured brightness values reflect this wider range. This value represents the average brightness of the pixels in that region, with higher pixel intensity values indicating higher brightness. The brightness values for the five squares were averaged to compute the final average brightness for each channel, with each channel representing a different experimental condition. The macro saved the results in a table that included the image name, the condition represented by each channel (e.g., “PLLA OM—Col10”), and the corresponding average brightness for each channel. The data was exported as a CSV file for further analysis. Appendix E contains the detailed Macro script.

### 2.6. Statistical Analysis

To assess the statistical significance of the differences observed among the groups, we employed a one-way analysis of variance (ANOVA). This approach enabled us to determine whether any of the mean values for the measured outcomes varied significantly across the experimental groups. For each test, we utilized a sample size of n = 3 biological replicate in 3 technical replicates, ensuring adequate statistical power for the analysis.

## 3. Results

**Figure 1:**
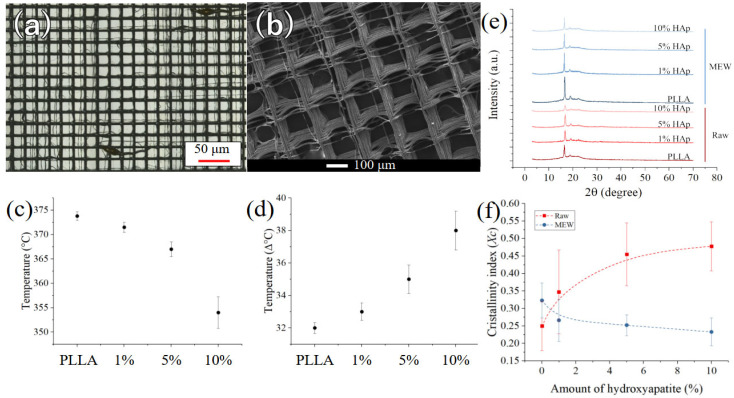
(**a**) Structure of the pure PLLA scaffold as observed at low magnifications, (**b**) structure of the pure PLLA scaffolds as observed at higher magnifications using SEM, (**c**) degradation temperature and (**d**) full-width at half-maximum of the degradation peaks of Figure A3 as a function of the amount of hydroxyapatite in the composite materials, (**e**) XRD results for the materials before (red) and after (blue) melt-electrowriting, (**f**) Crystallinity index for the different composites, following the equation presented in Section 2.

**Figure A1:**
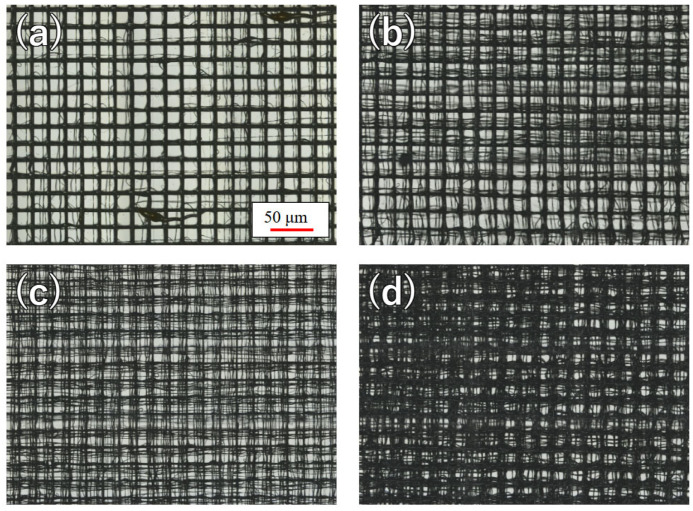
Morphology of the different scaffolds at low magnifications: (**a**), pure PLLA, (**b**), 1% wt. HAp, (**c**) 5% wt. HAp and (**d**) 10% wt. HAp.

The structure of the scaffolds is presented in Figure 1a, at low magnifications. Additional low-magnification images for all scaffold compositions are provided in Appendix A (Figure A1). For pure PLLA, the lattice appears to be regular, but a few defects such as PLLA droplets and misaligned fibers can be clearly observed. As the amount of reinforcement progressively increases, the defect density also increases, making the lattice structure barely visible for HAp concentrations of 10 wt.% (Figure A1d). The presence of HAp powder disturbs the polymer flux, reducing control over fiber positioning. Moreover, the addition of HAp powder increases the viscosity of the composite solution, further impeding precise fiber positioning during scaffold fabrication.

**Figure A2:**
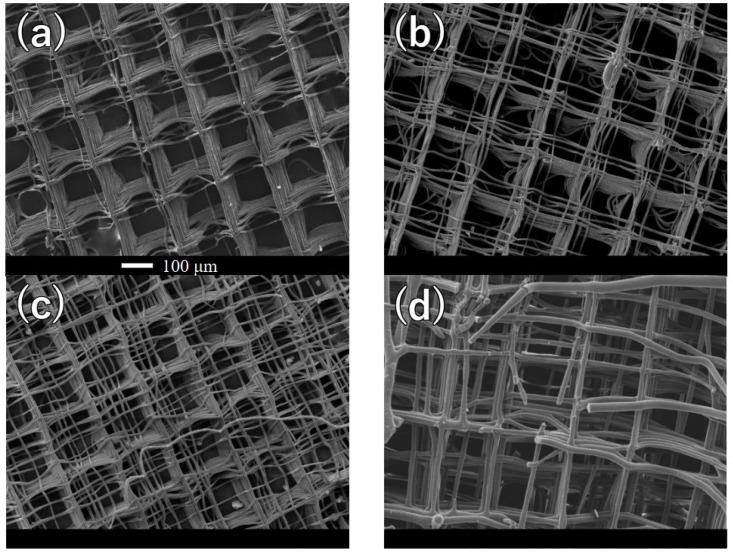
Morphology of the different scaffolds at higher magnifications: (**a**), pure PLLA, (**b**), 1% wt. HAp, (**c**) 5% wt. HAp and (**d**) 10% wt. HAp.

At higher magnifications (Figure 1b), the lattice of the scaffold, and in particular the characteristics of the fibers and the types of defects, can be observed more clearly. A complete comparison of high-magnification images can be found in Appendix A (Figure A2). While the pure PLLA scaffold appears to be almost completely regular, with the fiber layers well stacked on each other, when 1 wt.% HAp is added to the matrix, fibers from the last few layers do not properly stack (Figure A2b). This is caused by the gradient of temperature and electric field, as these fibers are the furthest from the building plate. This effect becomes even more pronounced when the amount of reinforcement reaches 5 wt.% (Figure A2c). For scaffolds containing 10 wt.% HAp, the increased viscosity of the solution results in a noticeable increase in fiber diameter, misalignment of fibers even near the building plate, and sudden filament interruptions (Figure A2d).

**Figure A3:**
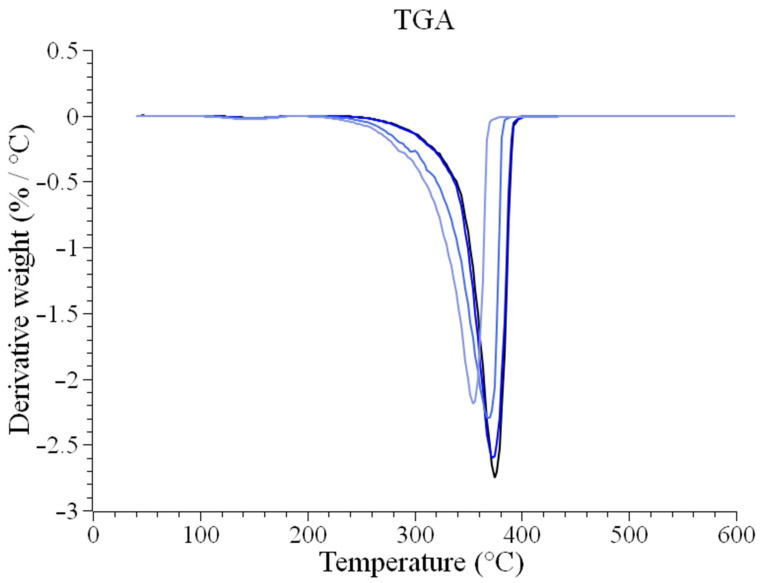
Derivative weight loss % as a function of temperature, for the four different scaffolds.

To understand the thermal stability, the scaffolds were analyzed using TGA. The graph provided in Figure A3 of Appendix B shows the derivative weight %/°C as a function of temperature, ranging from room temperature to 600 °C. It can be observed that the degradation temperature, which is close to 380 °C for pure PLLA, progressively shifts to lower values in the composite materials as a function of the HAp content, reaching approximately 350 °C for the highest reinforcement concentration (10 wt.%). This is due to the influence of HAp on the thermal degradation of PLLA, as it catalyzes decomposition reactions and accelerates the onset of degradation.

Figure 1c shows the trend of the degradation peak position and Figure 1d its full-width at half-maximum, as a function of HAp concentration. As the amount of reinforcement increases, the peak temperature progressively decreases and the width of the peak increases. This is caused by the incorporation of HAp into the PLLA matrix, which disrupts the polymer chain mobility and accelerates the degradation process. Additionally, the presence of HAp particles may act as nucleation sites for degradation, leading to a broader distribution of degradation temperatures.

To further elucidate the crystalline structure and phase composition, X-ray diffraction (XRD) analysis was conducted on both the composite materials and the MEW scaffolds. The diffraction patterns presented in Figure 1e exhibit distinct differences and trends between the two procedures, but the characteristic bands associated with hydroxyapatite could not be detected under the utilized experimental conditions, due to the low relative intensity and concentration. Figure 1f shows the trends in crystallinity index of the PLLA matrix as a function of the amount of HAp, for both the composites and the MEW scaffolds.

The pristine PLLA material exhibits a relatively low crystallinity index that rapidly increases with the incorporation of HAp during solvent evaporation. This phenomenon arises from the process dynamics: the dispersed HAp particulates serve as nucleation sites for PLLA crystallites, which have the opportunity to slowly grow. In contrast, in the context of melt-electrowriting (MEW), material solidification occurs rapidly, and the presence of HAp particulates hinders the crystallization process, acting as structural defects and discontinuities.

**Figure 2:**
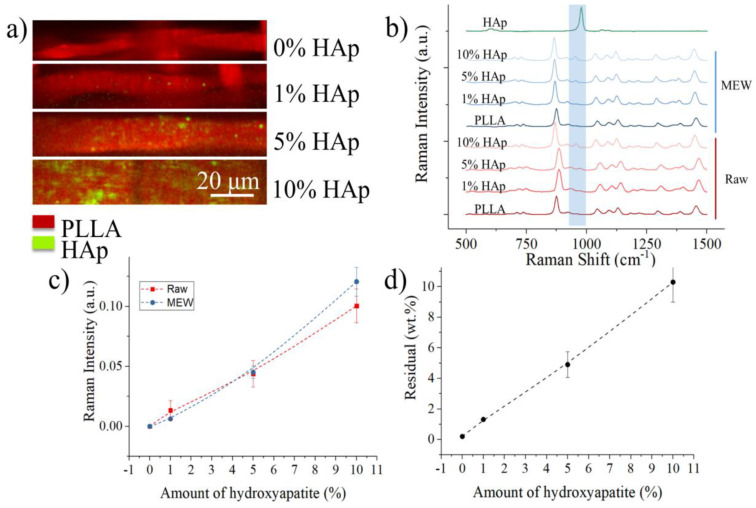
(**a**) Raman imaging of fibers as a function of HAp content, (**b**) average Raman spectra of the various materials, before and after MEW, compared with the spectrum of pure HAp, (**c**) relative Raman intensity of the hydroxyapatite PO_4_^3−^ band with respect to the main band of PLLA, as a function of theoretical content of HAp, (**d**) residual weight % after TGA (up to 700 °C) as a function of the theoretical content of HAp.

Raman imaging of the scaffolds was used to qualitatively determine the concentration and distribution of ceramic particulate inside the polymeric matrices. Figure 2a shows the chemical maps obtained on the four different surfaces, with red marking the presence of PLLA and green indicating HAp particles. At the lowest concentration, the particles appear to be well distributed inside the polymeric matrix, while as the concentration increases, they tend to accumulate at specific locations. This is true in particular for the sample containing 10% wt. of Hap particulate, and it reflects in the overall lower printability for this composite material.

The average Raman spectra of the different composites and scaffolds (Figure 2b) mainly features the bands related to the PLLA matrix, with the main peak related to hydroxyapatite, situated at about 960 cm^−1^ and related to PO_4_^3−^ vibrations, is barely visible at a concentration of 1% wt. and 5% wt. The complete peak assignation for both PLLA and HAp is presented in Table A1, along with some relevant literature references [51,52,53,54]. The bands whose assignment is indicated with an additional * are not normally present in PLLA according to literature data, these could be bands from additives. Figure 2c shows the relative Raman intensity of the band located at 960 cm^−1^ and related to Hap with respect to the band located at ~870 cm^−1^ and associated with C-COO stretch vibrations in PLLA. For both the composite materials and the MEW-printed scaffolds the intensity of the HAp band increases with the concentration, as expected, and the values of the two sets of samples are comparable, indicating that there is a relatively uniform distribution of ceramic reinforcement in the matrix both before and after printing.

The intensity of the Raman signal of HAp is clearly correlated to the concentration, but Raman spectroscopy is not an ideal quantification tool for composite materials. In order to provide a more accurate quantification, we considered the residual weight after TGA conducted up to 650 °C (Figure 2d) which shows a trend comparable with that of Raman spectroscopy and residual weights very close to the theoretical values, indicating that the process achieved a good control on the chemical composition of the scaffolds.

**Figure A4:**
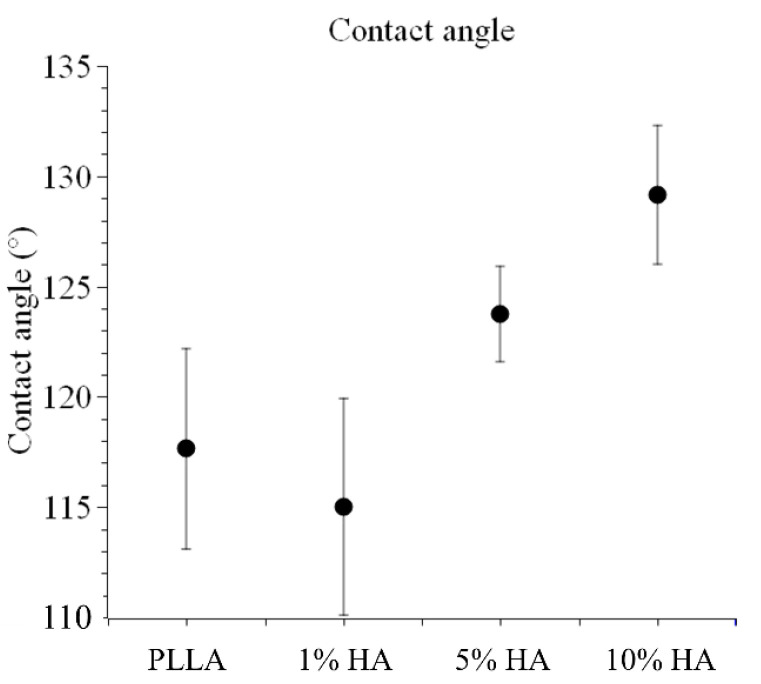
Contact angle as measured on the different scaffolds.

Contact angle measurements are presented in detail in Appendix D (Figure A4). Tests performed on the four scaffolds indicate that the presence of HAp increases the hydrophobicity of PLLA, going from about 115° for the pure polymer to approximately 130° when 10% wt. of HAp is added as a reinforcement. Although hydroxyapatite is intrinsically hydrophilic, the observed increase in contact angle can be attributed to a combination of factors. In the MEW-printed scaffolds, the PLLA matrix dominates the fiber surface, while HAp particles may be partially exposed depending on their distribution and incorporation during printing. Furthermore, the micro- and nano-scale surface topography induced by MEW, including fiber diameter, spacing, and roughness, amplifies the apparent hydrophobicity via the Cassie–Baxter effect. Thus, the progressive increase in contact angle is likely a result of alterations in scaffold morphology (Figure 1a,b) rather than the chemical nature of HAp alone. Notably, pre-wetting the scaffolds in culture medium prior to cell seeding facilitates protein adsorption on the surface, creating a biologically active interface that supports cell adhesion despite the high static contact angles.

**Figure 3:**
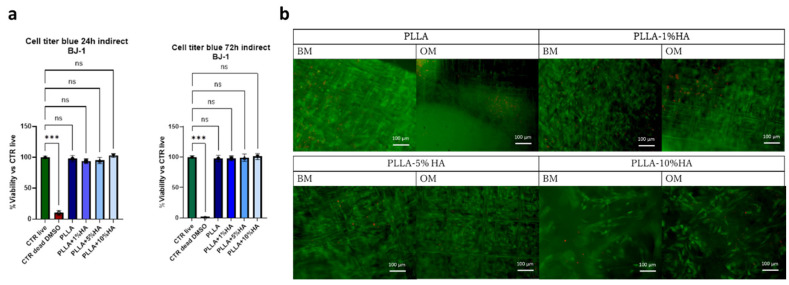
(**a**) Cell viability of BJ-1 cells according to ISO 10993 for indirect test using PLLA-HAp extracts for 24 h in Cell culture medium. Cell titer blue results are shown as % living cells relative to control (CTR live) that represent cells no treated with PLLA extract (n = 3; ANOVA one-way test using untreated cells as control). Bars show BJ1 exposed to PLLA alone or PLLA modified with HAp or not for 24 h (**left**) or 72 h (**right**), respectively, dark blue PLLA, blue PLLA + 1%HA, Light Blue PLLA + 5% HA, pale blue PLLA + 10%HA, green No material exposure (CTR Live) living cell control and (CTR dead DMSO) dead cell control; n = 3 replicates. NEJM ns 0.12, * *p* < 0.033, ** *p* < 0.002, *** *p* < 0.001. (**b**) Fluorescence microscopy analysis of hNC cells Live & Dead staining of PLLA samples modified with 1% HA, 5% HAp and 10% HAp exposed to Basal Medium (BM) or Interphase pro-osteochondral medium (OM). Green channel represents the calcein and living cells, red channel represents the dead cells and cell nuclei. Scale bar 100 µm.

For the toxicity study, the BJ1 cell line, one of the conventional cell lines used in ISO 10993 standards, was used. The analysis was performed indirectly; specifically, the cells were seeded on a plate and not directly on the scaffolds. Simultaneously, the scaffolds were placed in the medium. After 24 h, the medium in contact with the scaffolds was added to the cell culture medium. From the analysis, performed after 24 and 72 h, which also included control groups such as the dead cell group and the group of cells that had never been exposed to any medium in contact with scaffolds (CTR live), we found that none of the materials is toxic or affected cellular metabolic activity (Figure 3a) In fact, no statistical difference is observed between the study groups PLLA, PLLA + 1% HA, PLLA + 5% HA, PLLA + 10% HAp and the CTR live group, unlike the DMSO-dead cells control.

To evaluate the biological effects of hydroxyapatite (HA) on cells with strong cartilage repair potential and phenotypic plasticity toward interphase layer, human nasal chondrocytes (hNCs) were chosen as the cellular model. Once hNCs reached ~70% confluence, they were seeded onto composite scaffolds and cultured for 21 days in either Basal Medium (BM) or Osteogenic Medium (OM) to induce differentiation. The study aimed to assess whether HA-modified PLLA scaffolds exert cytotoxic or protective effects on hNCs. After 21 days of culture, robust cell growth and high viability were observed on all scaffold types, as confirmed by strong Calcein-AM fluorescence staining. (Figure 3b). Minimal cytotoxicity was detected, as demonstrated by the low presence of ethidium homodimer-positive cells, confirming limited cell mortality. Furthermore, OM did not appear to introduce additional cytotoxic effects but rather influenced the cell response in relation to HAp presence and concentration.

As observed in the figure, chondrocytes preferentially adhered to and aligned along the PLLA filaments, adopting a highly ordered orientation consistent with morphology-driven cell organization. This structural arrangement likely reflects the synergistic effects of scaffold architecture, HAp incorporation, and the differentiation medium, which together modulate cytoskeletal organization, cell–matrix interactions, and potentially direct lineage-specific differentiation

**Figure 4:**
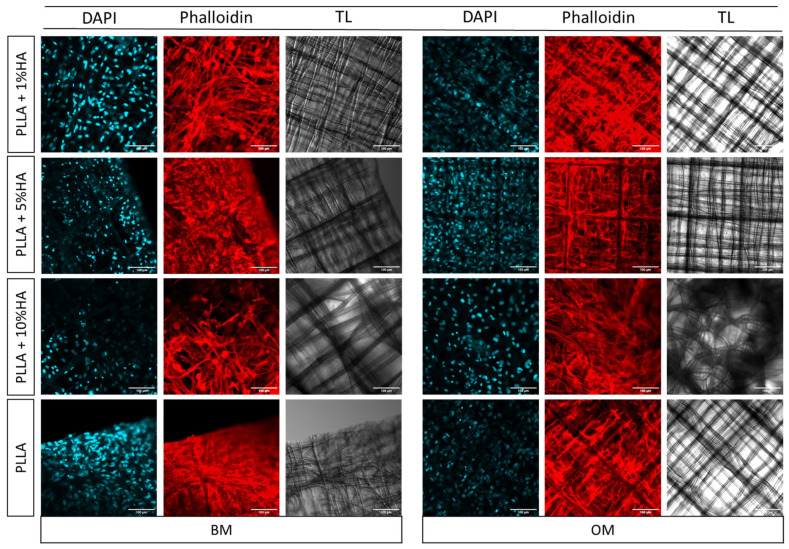
Confocal microscopy analysis cell morphology at day 21 of hNCs cells stained with Phalloidin. hNCs were seeded on PLLA samples modified with 1%, 5% and 10% hydroxy apatite in Basal Medium (BM) or Osteogenic Medium (OM). Red channel represents the cell morphology of hNCs stained with phalloidin, blue channels shows nuclei counter staining by DAPI and in gray is the scaffold acquired with transmission light. Scale bar 100 µm.

To thoroughly assess the cellular morphology of nasal chondrocytes and the impact of various conditions such as PLLA modification with HAp and the use of both BM and OM for the chondral-to-osseous interface we stained the cells with phalloidin and DAPI. This allowed for a clearer visualization of their arrangement on the scaffold structures (Figure 4). As observed from the live and dead images (Figure 3b), the cells exhibited organized adhesion to the scaffold, aligning along its structure in a well-ordered manner. Notably, in OM, cellular growth was more pronounced, with a significantly larger colonized scaffold surface compared to the corresponding growth observed in BM. This suggests that OM may enhance cell proliferation. Interestingly, from a morphological standpoint, in BM, the hNCs tended to aggregate more and had a rounder-like shape on PLLA-modified HAp 1% and 5%. In contrast, the same cells seeded on PLLA showed less round-shape morphologies. Nasal chondrocytes seeded on PLLA-10% HAp resulted to be more numerous and elongated, regardless of whether they were in the BM or OM.

Finally, we evaluated how the modification with HAp on PLLA, influence extracellular matrix (ECM) deposition by hNCs, we therefore perform staining to detect calcium deposits (specific of mineralized matrix) or sulfated glycosaminoglycan (sGAG)-positive ECM (specific for cartilage). Bone mineralization was assessed using Alizarin Red staining, that binds specifically to calcium ions, forming an orange-red complex that highlights areas of mineralization so we used to mark all mineralized areas on the entire scaffolds and subsequently quantified with a spectrophotometer.

**Figure 5:**
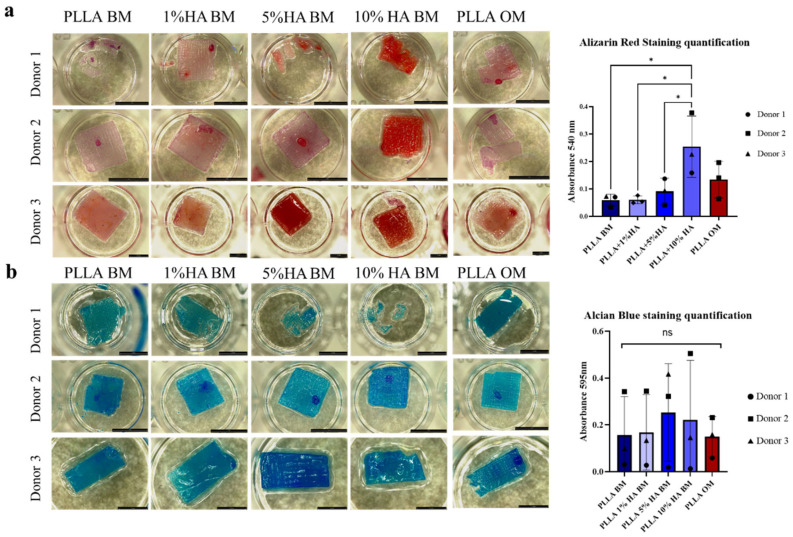
Characterization of the extracellular matrix deposed by hNCs cultured on PLLA samples modified with 1%, 5% and 10% hydroxyapatite for 21 days in Basal Medium (BM) or Osteogenic medium (OM). hNCs. (**a**) Alizarin Red staining for mineralization acquired with Leica stereoscope at different magnification, Alizarin red staining quantification dissolved in Cetylpyridinium Chloride. Blue bars show cells in BM, Red bar shows cells in OM. (**b**) Alcian Blue staining for Glycosaminoglycan deposition acquired with Leica stereoscope. Alcian Blue quantification Blue bars shows cells in BM, Red bar shows cells in OM. Scale bar 100 µm. Two-Way ANOVA GP:0.1234 (ns), 0.0332 (*).

In Figure 5a, it can be observed that there is a greater positivity to Alizarin Red in the PLLA-10% HAp group cultured in BM compared to the control group of PLLA cultured in OM. For the other groups, PLLA, PLLA + 1% HA, and PLLA + 5% HAp in BM, a donor-dependent variation was observed. Finally, we quantified Alizarin Red with a spectrophotometer using a wavelength of 540 nm specific for alizarin red dissolved in Cetylpyridinium Chloride. Results reported Figure 5a right confirmed the pro-osteogenic effect of HAp at the highest percentage (that were higher than the one induced by the OM) specifically in the group PLLA BM + 10% HAp (*, 0.0319 vs. PLLA BM, * 0.0319 vs. PLLA BM 1% HA, * 0.0491 vs. PLLA BM+ 5% HA).

Cartilaginous matrix production was assessed using Alcian Blue staining, that binds to sGAGs. It binds specifically to the carboxylated and sulfated groups of these polysaccharides, staining them in shades of blue. The results of Alcian Blue staining in Figure 5b, show that all constructs were positively stained for sGAG and that intensity of straining strongly varied among the hNCs. The spectrophotometric analysis corroborated this trend however not statistically significative (ns all group vs. PLLA BM). These results indicate that cartilage matrix deposition was not impaired by the presence of HAp in the scaffold nor by the presence of osteogenic factor in the culture medium (ns PLLA OM vs. PLLA BM).

**Figure 6:**
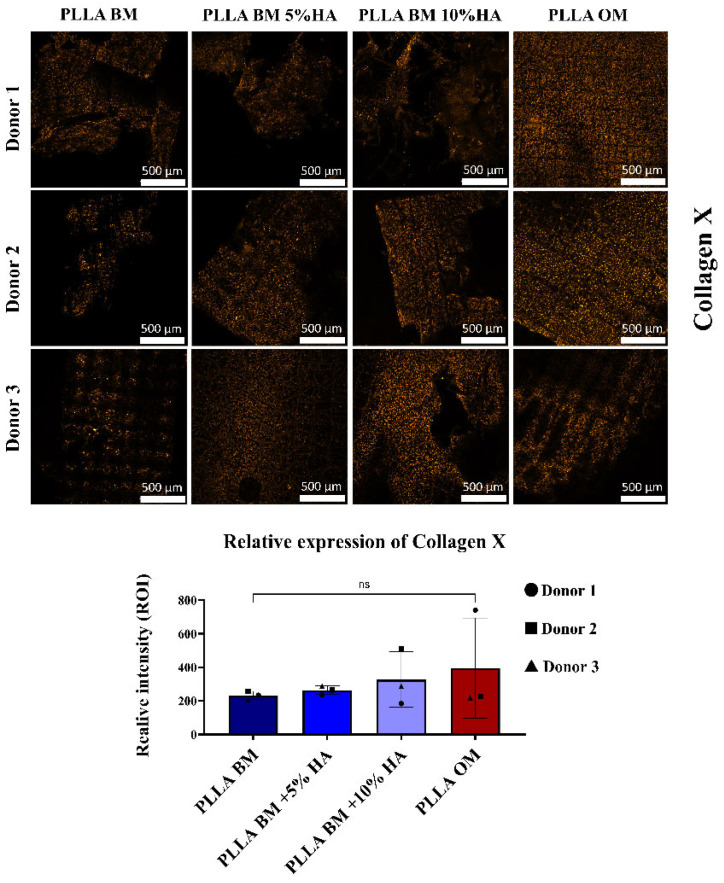
Immunofluorescence analysis of matrix biomarkers associated with cartilage hypertrophic Collagen X orange in human nasal chondrocytes (hNCs) at day 21. Cells were seeded on PLLA scaffolds modified with 1%, 5%, and 10% hydroxyapatite (HA) in basal medium (BM) or on unmodified PLLA scaffolds cultured in osteogenic medium (OM). Images were obtained from three technical replicates and three biological replicates (scale bar: 500 µm). On the right panel, the relative fluorescence intensity analysis quantifies protein expression from five points images of different donors. Blue bars represent fluorescence intensity for hNCs seeded on PLLA and HA-modified PLLA scaffolds cultured in BM, whereas the red bar corresponds to hNCs seeded on unmodified PLLA scaffolds in OM, acting as a reference condition. Additional confocal microscopy results of cells stained with DAPI and phalloidin at day 21 are presented in Appendix F, Figure A5.

To assess the impact of hydroxyapatite (HAp) incorporation into PLLA scaffolds on chondrocyte hypertrophy, we focused our analysis on the expression of Collagen X (COL10), a well-established marker of hypertrophic differentiation. In this part of our study, we specifically examined the effects of 5% and 10% HAp concentrations. As shown in Figure 6, COL10 expression was markedly upregulated in human nasal chondrocytes (hNCs) cultured on PLLA scaffolds modified with 5% and 10% HAp, even under basal medium (BM) conditions. The osteogenic medium (OM) control, used to induce hypertrophic and osteogenic differentiation, resulted in the highest levels of COL10 expression. However, a similar upregulation trend was observed in the 10% HAp-modified PLLA group cultured in BM, suggesting that higher HAp content alone may partially mimic or potentiate the effects of osteogenic cues. Despite donor-to-donor variability in expression levels, this response was consistent in pattern compared the group PLLA BM. Indeed, minimal COL10 expression was observed in hNCs seeded on unmodified PLLA scaffolds under BM conditions, supporting the hypothesis that HAp plays a key role in triggering hypertrophic signaling. Interestingly, increased COL10 expression in HA-modified scaffolds was spatially associated with an interfacial layer observed between the cells and the scaffold surface. This interface may represent a transitional zone formed through cell–material interactions or localized matrix remodeling, potentially enriched in HAp and mechanically distinct from the bulk scaffold.

## 4. Discussion

This study demonstrates the significant influence of HAp content on the morphology, thermal stability, crystallinity, and surface properties of PLLA scaffolds obtained by melt-electrowriting and their capacity to modulate proliferation and matrix deposition by nasal chondrocytes. These findings are essential for guiding the design and fabrication of composite scaffolds for osteochondral tissue engineering, where achieving an optimal balance of mechanical strength, biocompatibility, bioinductive potential, and controlled degradation is critical for clinical success.

The addition of HAp significantly impacted the scaffold morphology (Figure 1a,b). Pure PLLA scaffolds exhibited lattice structure. However, increasing HAp content resulted in a progressive decrease in structural order, with higher concentrations leading to barely visible lattice patterns and increased defect density. This disruption can be attributed to the hampered polymer flow and fiber positioning due to the presence of HAp particles and the increased viscosity of the composite solution, a hypothesis that is also supported by the changes in thermal property and crystallinity.

TGA revealed a decrease in the degradation temperature of PLLA with increasing HAp content (Figure 1c). This indicates a catalytic effect of HAp on the thermal decomposition of PLLA, accelerating the degradation process. Additionally, the broadening of the degradation peak (Figure 1d) suggests a wider distribution of degradation temperatures, potentially due to the presence of HAp particles acting as nucleation sites. XRD analysis (Figure 1e) showed that HAp incorporation significantly increased the crystallinity of PLLA in composite scaffolds fabricated by solvent evaporation. This is attributed to the dispersed HAp particles acting as nucleation sites for PLLA crystallite growth during solvent evaporation. Conversely, HAp hindered the PLLA crystallization process in MEW-fabricated scaffolds due to the rapid solidification and the presence of HAp as structural defects. Raman imaging (Figure 2a) confirmed the presence and distribution of HAp particles within the PLLA matrix. While well-distributed at lower concentrations, HAp tended to agglomerate at higher concentrations (10% wt.), potentially impacting printability. However, the relative Raman intensity of the HAp band increased proportionally with concentration (Figure 2c), suggesting a relatively uniform distribution of ceramic reinforcement both before and after printing.

Similarly to most ceramic-reinforced polymers, optimizing properties in PLLA scaffolds becomes complex with increasing HAp content. This is because additional reinforcement degrades structural properties. In melt-electrowriting (MEW), this effect is amplified by the unique production technique. The needle extruder, even with relatively small particles, can disrupt polymer flow and cause blockages (occlusions). Additionally, MEW’s rapid solidification process further impacts properties like crystallinity. Pure PLLA exhibits less of this effect due to its inherently fast crystallization rate. However, the presence of HAp disrupts solidification by acting as local defects. This effect is particularly pronounced with nanometric powders due to their high surface area, leading to decreased crystallinity and overall increased fragility.

From a biological and toxicological perspective, as shown in Figure 3, PLLA and its modifications with HAp are non-toxic. This was expected since these materials are commonly used in biomedical applications. Although PLLA is a polymer with inherently hydrophobic properties, as observed in Appendix D Figure A4, the addition of HAp gradually increased the contact angle, suggesting an increase in hydrophobicity. This could potentially hinder cell adhesion. However, considering the nature of HAp, the increased hydrophobicity is most likely a more macroscopic effect caused by the altered morphology of the scaffolds. Based on the HAp distribution analysis (Figure 2a, Raman), we believe that the incorporation of HAp into PLLA has enhanced the microtopography, likely promoting cell adhesion.

Furthermore, all scaffolds were pre-treated by immersion in culture medium for one hour prior to cell seeding. We therefore believe that the synergistic effect of the material’s surface characteristics and the pre-wetting process played a crucial role in overcoming the hydrophobicity issue typically associated with the anhydrous material.

Additionally, in this study, we aimed to evaluate the effect of HAp on nasal chondrocytes, i.e., cells with remarkable cartilage repair capacity [14,15,55,56] and ability to differentiate into different cell types including osteoblast once exposed to specific environment [41,57]. In our study, we sought to understand whether a material like PLLA, printed with different architecture, could influence the cellular phenotype. Furthermore, we hypothesized that modifying with hydroxyapatite could influence the cellular phenotype by promoting a more osteochondral hypertrophic direction. Hydroxyapatite is one of the most commonly used materials for scaffold modifications due to its well-tolerated biocompatibility by tissues and the body, without causing adverse immune effects. As reported Yang et al. [58], in which showed that hydroxyapatite (HAp) enhances osteogenic differentiation by modifying surface topography, by promoting the differentiation of human bone marrow mesenchymal stem cells (hBMSCs) into osteoblasts, thus improving bone regeneration potential. As well Liu et al. [59] showed that nanophase hydroxyapatite (nHAP) enhances osteogenic differentiation by significantly increasing the expression of osteogenic markers such as Collagen I, osteocalcin, and osteopontin in bone marrow-derived mesenchymal stem cells (MSCs), contributing to improved bone formation and cell proliferation.

Therefore, in our study, we investigate the osteoconductive potential of hydroxyapatite (HA) in poly(L-lactic acid) (PLLA) to enhance cell adhesion and create an optimal environment for bone regeneration. Based on this, we aim to demonstrate that modifying PLLA with HAp could generate a scaffold capable of supporting cell integration for osteochondral defect regeneration evaluating the comparison to a pro-osteogenic stimulus.

From the morphological analysis in Figure 4, where we evaluated cell shape on various constructs, we observed that cells in the pro-osteochondral/osteogenic differentiation medium were much more numerous. This phenomenon could be attributed to the presence of supplements in the OM, such as ascorbic acid, beta-glycerophosphate, and dexamethasone, which are not present in the basal medium. In the BM group, cells tended to aggregate into clusters, a phenomenon also observed during Alcian blue staining, a histological dye primarily used to stain glycosaminoglycans. This aggregation could be due to the natural tendency of chondrocytes to cluster together, suggesting that in the absence of stimuli, the chondrocytes nature facilitates this type of morphology, even after 21 days.

Finally, after 21 days of culturing nasal chondrocytes on PLLA- HAp modified material and on PLLA only in a pro-ostochondral stimulus (OM) as differentiation control, we aimed to understand how modification with HAp at different percentages could influence either the maintenance of the chondrocyte phenotype or potentially promote a more hypertrophic osteoblastic phenotype. The Alizarin Red analysis (Figure 5) revealed a clear trend toward mineralization primarily in the PLLA + 10% HAp group. In the other groups, only limited mineralization was observed, mainly at localized points on the scaffold where cell condensation was higher. However, this minimal positivity was not reflected significantly in the spectrophotometric analysis.

Interestingly, Alcian Blue staining (Figure 5) showed that in BM group lacking TGFβ and other growth factors cells aggregated and produced more ECM. After 21 days, NCs in BM showed greater staining than the OM control, especially in the PLLA-10% group, suggesting dual production of mineralized and cartilage matrix. However, this remains controversial due to the complex role of HAp and chondrocyte hypertrophy. The literature is divided: some studies report no hypertrophic effect of HAp on MSCs and chondrocytes [60], while others show that in vivo, HAp promotes hypertrophic chondrocyte differentiation into osteoblasts in a tibial fracture model [61]. Similarly, a study using silk fibroin, chitosan, and nanoHAp observed hypertrophy by enhancing chondrocyte growth and proliferation [62].

Collagen type X (COL10) was selected as a primary molecular marker in this study due to its critical role in chondrocyte hypertrophy and endochondral ossification (Figure 6). Unlike collagen type II, which is predominantly associated with the early stages of chondrogenesis, COL10 is expressed specifically by hypertrophic chondrocytes during the maturation phase of cartilage, where it contributes to matrix remodeling and mineralization. Its expression marks the transition zone between cartilage and bone, making it a key indicator of osteochondral differentiation. In the context of tissue engineering, the ability to induce COL10 expression is particularly relevant for the regeneration of the osteochondral interface, a complex zone that requires the coordinated development of both cartilaginous and osseous tissues [63].

In this study, we observed an upregulation of COL10 expression in scaffolds containing 5% and 10% hydroxyapatite (HAp) compared to PLLA alone, suggesting that HAp plays a pivotal role in promoting chondrocyte hypertrophy and the early events of mineralization. Notably, the 1% HAp group was excluded from in-depth analysis due to the absence of discernible differences in COL10 expression or mineral deposition relative to the unmodified PLLA scaffolds. This decision allowed us to focus on the concentrations that demonstrated a more pronounced biological response, enabling a more precise evaluation of the dose-dependent effects of HAp on osteochondral maturation. The enhanced COL10 expression observed in the higher HAp content scaffolds, particularly when cultured in differentiation medium over 21 days, supports the hypothesis that HAp incorporation not only enhances the bioactivity of PLLA but also promotes osteochondral lineage commitment.

This effect may be attributed to the biochemical influence of HAp [64], as the gradual release of Ca^2+^ and PO_4_^3−^ ions can activate signaling pathways such as CaSR–MAPK and Wnt/β-catenin [65] as observed in several other cell type like bone marrow stem cells, adipose stem cells, which are known to drive chondrocyte hypertrophy and COL10 expression, consistent with previous observations by Wang et al. [66].

In line with our findings, and based on published studies, we would expect similar behavior in other cell types when cultured on PLLA–HAp scaffolds.

Collectively, these findings underscore the importance of scaffold composition in directing cellular behavior for prompting the maturity of engineered osteochondral tissues. The data also suggest that HAp concentrations of 5% and 10% may represent an optimal range for promoting the formation of a transitional tissue phenotype conducive to interface regeneration. Further studies are underway to better understand how the architecture and HAp concentration are actually influencing the cellular phenotype.

Current research on osteochondral regeneration has largely focused on hydrogel-based systems, which offer advantages in terms of cell encapsulation and biocompatibility. However, hydrogels are typically printed at lower resolution compared to melt-electrowritten or conventional scaffolds, and they often exhibit limited chemical and temporal stability, which can compromise their structural integrity during in vitro culture or after implantation [67].

Another major limitation of hydrogel-based constructs is their mechanical weakness, which is insufficient to support load-bearing tissues such as cartilage and subchondral bone. To address this issue, hydrogels are frequently reinforced with synthetic polymers such as PLA or PCL [68]. While this reinforcement improves mechanical stability, it can reduce bioactivity or introduce challenges in controlling cell–material interactions. Furthermore, much of the literature in osteochondral tissue engineering focuses on the use of stromal cells [67], which are relatively easy to isolate and expand but exhibit several limitations, including donor site variability, lower chondrogenic potential, and limited ability to form a stable hypertrophic interface. In contrast, we used nasal chondrocytes (hNCs), which have several advantages: they are readily accessible, display high proliferative capacity, maintain robust chondrogenic potential even after expansion, and are less affected by donor age. Additionally, nasal chondrocytes have been shown to generate a stable hypertrophic phenotype suitable for osteochondral interface formation, making them particularly attractive for applications targeting cartilage–bone regeneration [69].

In MEW, ordered micro-lattices give pore size and fiber orientation that steer osteogenesis; square pores around 100 µm were shown to maximize stiffness, seeding, and collagen/mineral deposition in MEW fibrous grids [70]. Electrospinning, on the other hand, typically yields nano-fibrous, highly porous structures with excellent surface area but less control over 3D strut placement. Hybrid “micro-/nano” architectures in PCL deliberately combine the two (MEW + electrospinning) to couple lattice mechanics with nanofiber bioactivity. In a MEW-plus-solution-electrospinning scaffold, adding HAp to the nano-layer increased water uptake and promoted osteoblast adhesion, proliferation and osteogenic differentiation. In PCL, this architecture appears to retain good mechanical properties. However, it is not possible to make direct speculations on whether a similar structure in PLLA would exhibit comparable behavior [71].

MEW with PCL-HAp has also pushed ceramic loading much higher when process parameters are tuned. Abdal-Hay et al. were able to incorporate 40 wt% into continuous, well-stacked fibers and yielded higher metabolic activity and increased OPN/ALP/COL1 expression vs. polymer-only controls, illustrating how strong mineral loading can amplify bioactivity but also negatively affect processability and toughness [72]. On the FDM/FFF side, PLA-HAp composites with ≥20 wt% HAp produced trabecular-bone-compatible compressive moduli and induced robust osteogenic differentiation of hMSC even without soluble osteogenic stimuli, while remaining immunologically inert to dendritic cells-showing the potential of higher ceramic fractions for osteoinductivity, albeit with the classic FDM trade-off of stiffer but less tough filaments [73]. Compared to FDM PLA-HAp, our MEW PLLA-HAp places greater emphasis on micro-architectural guidance (e.g., ~100 µm pores) with lower ceramic loading to gain bioactivity while limiting the crystallinity and degradation resistance loss we measured.

A key limitation of this study is its exclusively in vitro nature. Although the experiments were conducted with rigorous methodology, the controlled conditions of the culture environment cannot fully reproduce the complex biochemical, biomechanical, and immunological cues present in living tissue. For example, factors such as vascularization, mechanical loading, immune cell interactions, and systemic metabolic regulation can profoundly influence chondrocyte behavior and matrix remodeling processes that are not captured in a static in vitro system. Consequently, the upregulation of hypertrophic markers such as COL10 and the mineralization patterns observed here may differ in a physiological context. For this reason, in vivo validation is essential to determine whether the trends identified in this work translate into functional osteochondral tissue formation. Future studies should aim to address this by employing ethically designed animal models and minimizing animal use wherever possible, including the integration of ex vivo explant systems. Additionally, another limitation of this study is the absence of compression testing for the MEW-fabricated PLLA–HAp mesh scaffolds, which are intended to mimic and support the bone–cartilage interface. Although the present work focused on evaluating the feasibility and biological effects of HAp incorporation, mechanical performance is critical for this application, and the lack of such data may currently limit the translational potential of the scaffolds. Given that scaffold optimization is an iterative process, the feasibility and biological compatibility of HAp incorporation was assessed before proceeding to mechanical characterization. This stepwise approach ensures that only biologically promising formulations are subjected to mechanical testing, which is currently in progress. Ongoing studies are addressing this aspect to provide a more comprehensive assessment.

## 5. Conclusions

In this study, we have successfully engineered poly(L-lactic acid) (PLLA) scaffolds integrated with hydroxyapatite using melt-electrowriting technology. The crystallographic, thermal and spectroscopic characterization techniques employed confirmed effective HAp dispersion within the PLLA matrix. However, the integration of HAp influenced the physical properties of the scaffolds. HAp integration into the melting process reduced PLLA crystallinity and lowered its degradation temperature, which could impact the scaffold’s long-term stability and mechanical performance. Moreover, an increase in HAp content led to higher defect density and reduced scaffold toughness.

Our results demonstrated that scaffolds with up to 10% HAp content showed significant improvements in biological properties. Specifically, in vitro assessments using human nasal chondrocytes revealed enhanced cell adhesion, proliferation, and mineralization over time and unaltered capacity to produced cartilaginous matrix components. These improvements are crucial for the successful integration and function of the scaffolds in osteochondral tissue engineering. This suggests a trade-off between bioactivity and mechanical integrity.

Despite these challenges, the scaffolds’ enhanced bioactivity due to HAp incorporation highlights their potential for clinical applications. The precise control over scaffold architecture and composition achieved through MEW technology allows for the production of highly porous structures that can support cellular activities essential for tissue regeneration.

## Figures and Tables

**Figure A5:**
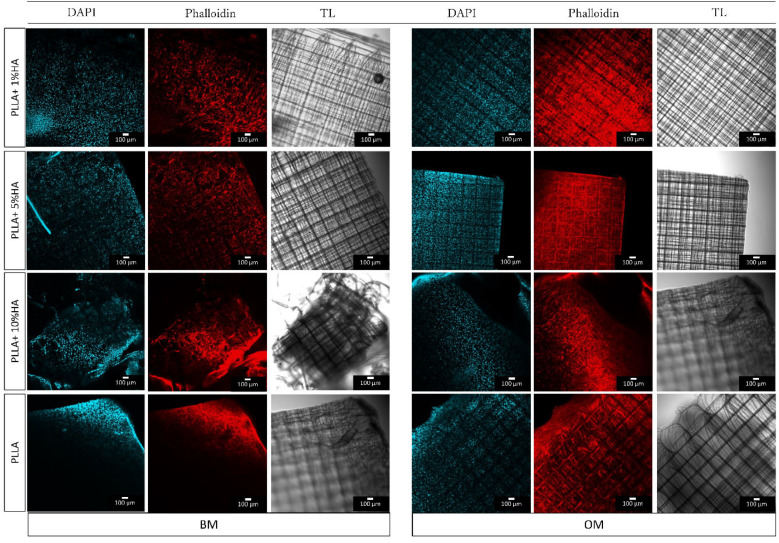
Confocal microscopy analysis cell morphology at day 21 of hNCs cells stained with Phalloidin. Lower magnification. Scale bar 100 µm.

## Data Availability

The data supporting the findings of this study are available from the corresponding author upon reasonable request.

## Abbreviations

The following abbreviations are used in this manuscript:

BM	Basal medium
BSA	Bovine serum albumin
CCD	Charge-coupled device
COL10	Collagen X
CTR	Control
DMEM	Dulbecco’s modified Eagle medium
DSC	Differential scanning calorimetry
DMSO	Dimethyl sulfoxide
FBS	Fetal bovine serum
HAp	Hydroxyapatite
hNCs	Nasal condrocytes
MEW	Melt-electrowriting
OM	Osteogenic medium
PBS	Phosphate-buffer saline
PLLA	Poly-l-lactic acid
ROIs	Regions of interest
SEM	Scanning electron microscope
TGA	Thermogravimetric analysis
XRD	X-ray Diffraction

## Appendix A. Morphology

This section presents the morphological analysis of the four different scaffolds: pure PLLA, 1 wt.% HAp, 5 wt.% HAp, and 10 wt.% HAp. Representative low-magnification laser microscope images are provided to illustrate the overall structure and surface features of the scaffolds (Figure A1). Additionally, high-magnification scanning electron microscope (SEM) images are included to examine the detailed microstructural characteristics of each composition (Figure A2).

## Appendix B. Thermogravimetric Analysis

The thermogravimetric analysis (TGA) results are presented in Figure A3 as derivative weight (%/°C) as a function of temperature, providing insights into the thermal degradation behavior of the scaffolds.

## Appendix C. Spectroscopic Characterization

The following table presents the assignments for the Raman bands observed in Figure 2b, identifying the characteristic vibrational modes of the scaffold components.

**Table A1:** Raman assignments for the bands observed in Figure 2b.

Band Position [cm^−1^]	Assignment	Material [51,52,53,54]
~1450	CH_3_ asymmetric stretch	PLLA
~1388	CH_3_ symmetric stretch	PLLA
~1365	CH deformation and CH_3_ symmetric deformation	PLLA
~1295	CH deformation	PLLA
~1220	COC asymmetric stretching	PLLA
~1180	COC asymmetric stretch	PLLA
~1127	CH_3_ asymmetric rocking	PLLA
~1095	COC symmetric stretch	PLLA
~1076	PO_4_^3−^ symmetric stretching mode	HAp
~1049	PO_4_^3−^ asymmetric stretching	HAp
~1044	C-CH_3_ stretch	PLLA
~960	PO_4_^3−^ symmetric stretching	HAp
~950	CC stretching and CH_3_ rocking	PLLA
~920	CC stretching and CH_3_ rocking	PLLA
~870	C-COO stretch	PLLA
~737	C=O deformation (in-plane)	PLLA
~708	C=O deformation (out-of-plane)	PLLA
~610	PO_4_^3−^ asymmetric bending	HAp
~592	PO_4_^3−^ asymmetric bending	HAp

## Appendix D. Contact Angle

The contact angle results for the various scaffolds are presented below, providing an evaluation of the surface wettability and hydrophilicity for each composition.

## Appendix E. Macro Script for Fijii ImageJ

// Get the list of files in the folder

fileList = getFileList(inputDir);

// Filter the file list to include only .nd2 files

nd2Files = newArray();

for (f = 0; f < fileList.length; f++) {

if (endsWith(fileList[f], “.nd2”)) {

nd2Files = Array.concat(nd2Files, fileList[f]);

}

}

// Check if any .nd2 files were found

if (nd2Files.length == 0) {

exit(“No .nd2 files found in the selected folder.”);

}

// Prepare the results table

run(“Clear Results”);

setResult(“Image Name”, 0, ““); // Ensures Results table starts fresh

// Loop through all .nd2 files

for (f = 0; f < nd2Files.length; f++) {

fileName = nd2Files[f];

// Open the .nd2 file using Bio-Formats

run(“Bio-Formats Importer”, “open=[“ + inputDir + fileName + “] autoscale color_mode=Default rois_import=[ROI manager] view=Hyperstack stack_order=XYCZT”);

// Get image dimensions and number of channels

dims = newArray(0, 0, 0, 0, 0); // Array to store [width, height, channels, slices, frames]

**getDimensions(dims** [0]**, dims** [1]**, dims** [2]**, dims** [3]**, dims** [4]**); // Populate dimensions array**

**nChannels = dims** [2]**; // Extract number of channels**

// Ensure channels are found

if (nChannels < 1) {

print(“No channels found in file: “ + fileName);

close();

continue;

}

// Increase the brightness of all channels by 50%

print(“Increasing brightness by 50% for: “ + fileName);

run(“Multiply…”, “value=1.5 stack”);

// Define positions of the five squares

xPositions = newArray(

(dims [0] - squareSize) / 2, // Center

250, // Top-left corner

dims [0] - squareSize - 250, // Top-right corner

250, // Bottom-left corner

dims [0] - squareSize - 250 // Bottom-right corner

);

yPositions = newArray(

(dims [1] - squareSize) / 2, // Center

250, // Top-left corner

250, // Top-right corner

dims [1] - squareSize - 250, // Bottom-left corner

dims [1] - squareSize - 250 // Bottom-right corner

);

// Analyze each channel

for (c = 1; c <= nChannels; c++) {

// Set the current channel

Stack.setChannel(c);

// Variable to store the total brightness for averaging

totalBrightness = 0;

// Analyze the five squares

for (i = 0; i < xPositions.length; i++) {

// Get the current position

x = xPositions[i];

y = yPositions[i];

// Create the square ROI

makeRectangle(x, y, squareSize, squareSize);

// Measure the brightness of the ROI

run(“Measure”);

mean = getResult(“Mean”, nResults - 1); // Get the mean brightness

// Add to total brightness

totalBrightness += mean;

}

// Calculate the average brightness of the five squares

averageBrightness = totalBrightness / xPositions.length;

// Add the results to the Results table

setResult(“Image Name”, nResults - 1, fileName);

setResult(“Channel”, nResults - 1, “Channel “ + c);

setResult(“Average Brightness”, nResults - 1, averageBrightness);

}

// Close the current image

close();

}

// Save the results to a CSV file

outputFile = outputDir + “Brightness_Results_Average.csv”;

saveAs(“Results”, outputFile);

print(“Analysis completed. Results saved to: “ + outputFile)
